# A Systematic Review of Augmented Reality in Health Sciences: A Guide to Decision-Making in Higher Education

**DOI:** 10.3390/ijerph18084262

**Published:** 2021-04-17

**Authors:** Carlos Rodríguez-Abad, Josefa-del-Carmen Fernández-de-la-Iglesia, Alba-Elena Martínez-Santos, Raquel Rodríguez-González

**Affiliations:** 1 Department of Psychiatry, Radiology, Public Health, Nursing and Medicine, Faculty of Nursing, University of Santiago de Compostela, 15704 Santiago de Compostela, Spain; carlosrodriguez.abad@usc.es (C.R.-A.); alba.martinez.santos@usc.es (A.-E.M.-S.); 2 Department of Pedagogy and Didactics, Faculty of Education Sciences, University of Santiago de Compostela, 15704 Santiago de Compostela, Spain; c.delaiglesia@usc.es

**Keywords:** augmented reality, higher education, health sciences, teaching-learning

## Abstract

The objective of this study was to investigate the usability of the augmented reality (AR) in higher education in the area of health sciences to describe what type of interventions have been developed, their impact on various psychopedagogical aspects of the students as well as the main advantages, disadvantages and challenges in incorporating AR in the teaching-learning process. A systematic review was carried out in the CINAHL, PsycINFO, MEDLINE, Web of Science databases and the Google Scholar search engine. The search was limited to original research articles written in English, Spanish or Portuguese since 2014. The quality of the selected articles (*n* = 19) was assessed using the Mixed Methods Appraisal Tool (MMAT). The applications and electronic devices used and the measurement instruments used were described. The use of AR made it easier for students to acquire skills, especially in courses with a high component of three-dimensional visualization, and positively influenced various aspects of the learning process such as motivation, satisfaction or autonomous learning. As an educational technological tool applied to higher education in health sciences, AR improves the teaching-learning process by influencing it in a multidimensional way.

## 1. Introduction

In recent decades, higher education has undergone an important methodological change because the curricular design is increasingly focused on the student. The use of information and communication technologies (ICT) in teaching-learning processes allows the teacher to facilitate student learning by adapting the contents and methodology to different rhythms and interests of the students [1]. The last report of the Department of European Projects of the National Institute of Educational Technologies and Teacher Training (INTEF), “EDUCASE Horizon Report: 2019 Higher Education Edition”, describes the six emerging technologies that will have a significant impact on higher education in the next five years (2019–2023), classifying them, based on implementation time, as “short, medium and long term.” Augmented reality (AR) is part of these technologies as a digital tool to be implemented in the short term, through mobile learning, and in the medium term, as part of mixed reality. The use of AR in the classroom could spread rapidly, since mobile devices (smartphones or tablets) are widely used by university students and can be used to disseminate didactic content based on this technology [2].

AR was defined in very different ways throughout its short existence, although the most widespread definition states that AR consists of a mixed environment that integrates digital information in a real environment [3]. Di Serio et al. (2013) highlight three properties of AR: they combine real and virtual objects in a real environment, align real and virtual objects with each other, and execute them interactively and in real time [4].

The use of AR in education is still in the early stages [3,4,5,6], despite having become more accessible as it does not require specialized equipment and can be easily used on mobile devices [3,5]

**Figure 1:**
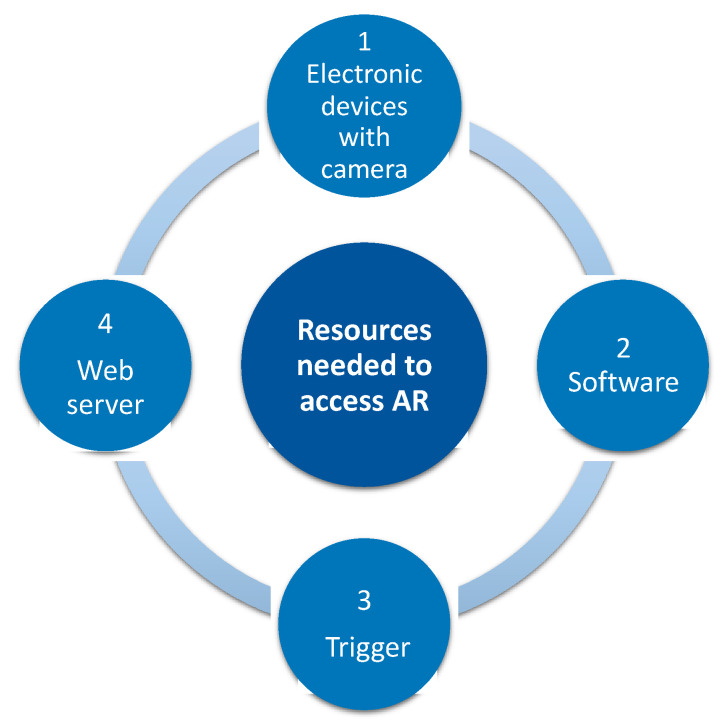
Resources needed to access AR. AR: augmented reality.

Figure 1 shows the resources needed to access AR content. In this case, four elements are needed. Firstly, an electronic device with a camera, such as tablet, smartphone, computer, smartwatch, smartglasses. Secondly, a software to integrate virtual content in the real word, together with a trigger to run AR content. Finally, a web server where the virtual information that we want to project on the real environment is stored [7,8].

The use of AR in higher education in the area of health sciences can contribute to improve the acquisition of skills, providing the student with a more authentic learning and a more personalized learning experience [9]. Likewise, it can contribute to make clinical practice workshops more realistic by facilitating the acquisition of competencies [10,11]. Therefore, there is evidence indicating a favorable impact of AR on medical training [12].

Our research presents the state-of-the-art of AR in health science higher education, describing the scholarly values of this technology. To facilitate decision-making by health science educators on specific didactic aspects in this field, a systematic review of the literature has been carried out with the aim of locating and integrating the main results of studies on this topic.

Specifically, the objective of this systematic review was to identify, evaluate and summarize the evidence conducted using augmented reality with health sciences students in higher education. This mixed method review aimed to answer the following questions:

(1) What kind of augmented reality interventions are used in health sciences higher education?
(2) What impact does the use of augmented reality have on the educational outcomes and skills of these students?
(3) What are the main advantages, disadvantages and challenges of this technology during the teaching-learning process?

## 2. Methods

### 2.1. Design

This review employed a data-based convergent synthesis design, a type of mixed method systematic review synthesizing evidence derived from quantitative and qualitative studies [13]. The Preferred Reporting Items for Systematic Reviews and Meta-Analyses (PRISMA) checklist was used to ensure an adequate quality standard design (see Supplementary Table S1) [14]. The PRISMA statement consists of 27 essential items checklist, widely used in health sciences, that help to guarantee the rigor and transparency of reporting of systematic reviews and a four phases flow diagram [15]. Thus, the PRISMA statement is a useful resource to perform systematic reviews and meta-analyses. No protocol was registered.

### 2.2. Search Methods

In order to explain the search process in detail, it is indicated by the initials what each author (see the first page for the full name) did to ensure the systematization of this step of the review, as well as the following steps. A systematic search approach of specific health and social science databases was independently performed by the authors CR and JCF from March 2020 to May 2020. These were CINAHL, PsycINFO, MEDLINE and Web of Science. A Google Scholar search was also performed. One of the authors’ expert knowledge (JCF) and previous articles were used to determine and refine the search terms and controlled vocabulary, when available. Keywords were searched in titles and abstracts, as well as in other fields when appropriate (see example CINAHL search strategy in Table 1). The search strategy involved a comparison of advanced and basic searches with appropriate combinations in different databases. The search was limited to original research studies written in English, Spanish or Portuguese and conducted from 2014 to the present. The search started on this date due to the rise of AR in recent years, especially since 2014, as indicated in the Horizon Report [16]. ProQuest RefWorks was used to manage the citations from the search results.

### 2.3. Search Outcomes

The search boundaries of the selected databases resulted in 1254 articles. After duplicates were removed, 1209 papers were screened according to following criteria outlined below.

**Figure 2:**
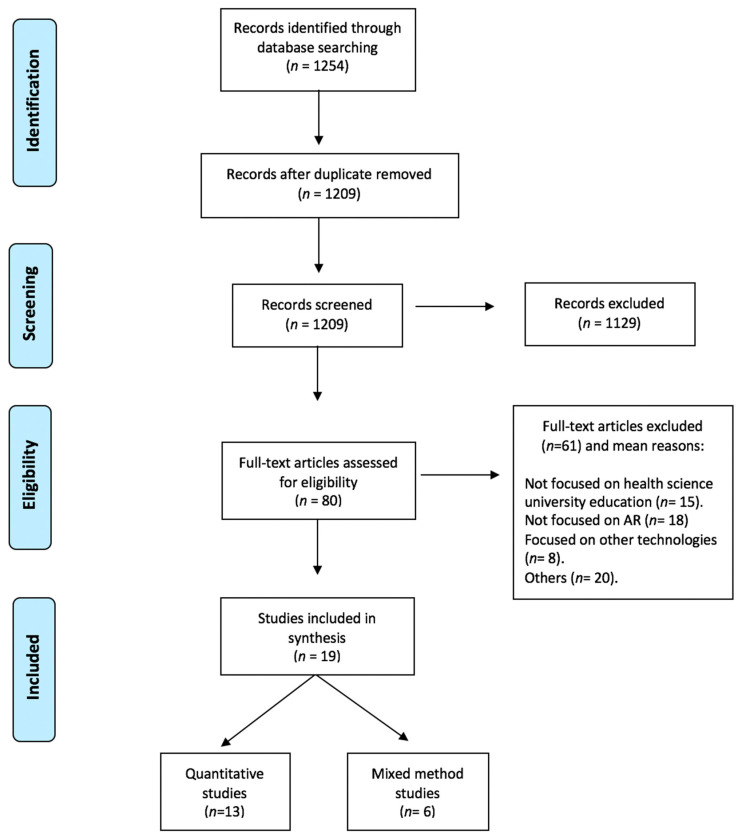
Flow chart of study screening process, according to PRISMA Framework.

Following the eligibility criteria, titles and abstracts were screened independently (CR & AM). For the stage two in the screening, the potentially relevant articles with full texts (*n* = 80) were assessed by the same authors. Any discrepancies or doubts during all the screening process were resolved by consensus within the other authors (JCF and RR). This stage led to 19 articles, after excluding 61 studies. Figure 2, following the PRISMA statement, details the methodological process used in this systematic review, including the reasons for the excluded full-text articles [17].

Regarding the eligibility, Inclusion criteria were: (1) articles published in peer-reviewed journals and (2) empirical studies that reported the use of AR in health science studies. Exclusion criteria were the following: (1) describe AR in areas of knowledge other than health sciences, (2) report AR at other educational levels other than higher education, (3) the technology used to support the teaching-learning process was not AR, (4) the objective of the study was not focused on AR, and (5) unable to source the full text paper reporting the original study.

### 2.4. Data Abstraction & Synthesis

The data extraction was completed by one author (CR) while other author independently checked the findings (AM). Once the data was extracted in text form, an ad hoc table was developed through consensus to categorize the data following the review aims. This abstraction was completed by the same authors (CR and AM) and included: author(s), year of publication, country, academic degree, academic year, course and sample size, aims and measuring instruments, results and MMAT rating.

The extracted data of quantitative studies (*n* = 13) and mixed method studies (*n* = 6) were subjected to a convergent thematic and narrative synthesis, in line with Hong et al. (2017) [13]. This type of mixed-methods synthesis made it possible to integrate the findings into the results section, maximizing the usefulness for reporting on practice [18].

### 2.5. Quality Appraisal

Quality appraisal was conducted independently by authors and final assessment for quality and the risk of bias was the product of the consensus. Systematic reviews with PRISMA require a critical appraisal of the included studies [19]. The Mixed Methods Appraisal Tool (MMAT) is a useful tool to assess the methodological quality of studies of different designs included in this systematic review [20]. Also, this critical appraisal tool has been used in many systematic reviews due to its checked utility [19]. Therefore, MMAT was employed to evaluate the selected studies in this review article, since this tool was useful for assess both qualitative and quantitative evidence [21]. This evaluation allowed us to identify studies of low methodological quality, and, since their exclusion is discouraged, the interpretation of research findings has considered the risk of bias/rigor.

The studies generally met the criteria of MMAT-checklist. Though, they did not consider some aspects: Descriptive quantitative studies mostly fail or did not allow responding to the MMAT 4.5 criterion “Is the statistical analysis appropriate to answer the research question?”. In the case of quantitative randomized controlled trials, they did not meet the MMAT 2.4 criterion “Are outcome assessors blinded to the intervention provided?”. In the analysis of the methodological quality of the quantitative non-randomized studies, it is not possible to respond to the MMAT 3.4 criterion “Are the confounders accounted for in the design and analysis?”. Lastly, two mixed-methods studies met all MMAT criteria, but the rest did not provide data to respond to the MMAT 5.3 criterion “Are the outputs of the integration of qualitative and quantitative components adequately interpreted?” (see Supplementary Tables S2–S5 for more details).

In summary, and according to the MMAT evaluation criteria, 72.2% of the articles (*n* = 13) met 75–100% of the MMAT checklist, representing high quality. 26.4% of the articles (*n* = 5) met 50–75% of the evaluated criteria, representing moderate quality. 5.4% of the articles (*n* = 1) met less than 50% of the evaluated criteria, representing low quality (Table 2).

## 3. Results

### 3.1. Characteristics of the Studies

The current review included 19 studies. Most of them took place in USA (*n* = 4), followed by Spain (*n* = 2), Netherlands (*n* = 2), Germany (*n* = 2) or Australia (*n* = 2). The remaining studies were conducted in UK, Denmark, Portugal, South Africa, Turkey, Brazil and Chile. No study was performed in Asia. Quantitative studies (*n* = 13) included questionnaires and surveys, while mixed-method studies (*n* = 6) utilized questionnaires and interviews about opinions or participants feedback about the experience. Study designs included randomized trials (*n* = 4), non-randomized trials (*n* = 4), descriptive studies (*n* = 5) and mixed method studies (*n* = 6). Sample sizes ranged from 4 to 880 students (see Table 2).

The degree in which the most AR experiences were developed was Medicine (*n* = 12) followed by Nursing (*n* = 3). The subjects in which AR was predominantly implemented were Human Anatomy (*n* = 12) followed by practical classes or Clinical Simulations (*n* = 3). Most of the studies were carried out with first (*n* = 7) or second-year students (*n* = 2). See Table 2 for more details.

### 3.2. Convergent Synthesis of Studies

The findings from the studies (*n* = 19) were synthesized and collated into three main themes which were:

#### 3.2.1. Technical and Pedagogical Usability of AR in Health Sciences Higher Education

- Technical usability involves techniques for ensuring a trouble-free interaction (readability and ease of use), and pedagogical usability aims to support the learning process; both terms should not be considered independently [22,23]. Taking this into account, contributions of AR in Health Sciences Higher Education in our review were diverse:
- In the development of skills oriented to decision-making and performance of practical procedures, especially in inexperienced individuals [24].
- In the creation of more realistic interactive learning spaces that promote and facilitate autonomous and collaborative learning, facilitating the acquisition of the skills provided in the study plans [11,12,25,26].
- In making learning more flexible, by allowing access to content at any time regardless of location [27].
- In the teaching-learning of human anatomy, since the three-dimensional vision provided by AR facilitates spatial understanding, and, therefore, the acquisition of knowledge [10].

Despite the numerous alternatives made possible by the use of AR as a didactic tool in Higher Education in Health Sciences, it is not implemented in a massive way [28]. Nevertheless, its application in this field has increased in the last few years due to the widespread use of mobile devices by students [6,11,25,29,30] as well as readability and ease of use.

##### Electronic Applications and Devices for the Creation and Visualization of Virtual Content

Most selected articles (*n* = 13) specified the software used to develop virtual content. The most used was *Unity* in combination with *Vuforia* (*n* = 6), showing advantages over others for its ease of use, its high graphic quality and that allows the creation of multiplatform applications. Applications such as *Metaio Creator* or *Aurasma* used by some authors [6,30] are currently no longer available. Others like ViewAR have an interface that allows inexperienced developers or developers with basic computer skills to create mobile AR applications. Few studies (*n* = 2) use AR mobile applications not developed specifically for their research, but instead were free applications, such as Anatomy 4D, that allowed students to interact with clipart images of the human body through mobile devices [29,30]. The application *Dynamic Anatomy*, available for *smartglasses* (Hololens), was developed expressly for the investigation of Bogomolova et al. (2020) and facilitates learning the dynamic anatomy of the ankle joint through its free access [31].

Table 3 shows the applications used, as well as the display devices and AR level. Among the electronic devices used to view virtual content, mobile devices (smartphones or tablets) were the most chosen to support the teaching-learning process (*n* = 10), followed by smartglasses (*n* = 5) and large monitors (*n* = 3). In one of the articles no data was indicated on the applications and devices used. Regarding AR levels, most articles (*n* = 11) used markerless technologies (image recognition).

##### Measurement Instruments

Various data collection instruments were used in the articles included in this systematic review. These were administered before, after or before and after the AR experiences. The instruments measured different parameters, such as the perception of the students with the AR experience, the degree of motivation that AR generates in the students, the cognitive load used in the learning process or the degree of acceptance of AR-based technology.

Specifically, to measure the perception of students with the AR experience, Agudelo et al. (2019) administered an ad hoc survey to the students with three dimensions: implementation and contributions to training, collaborative learning and motivation with the innovative methodology [25]. The validated questionnaire by Ferrer-Torregrosa et al. (2016) was based on metacognitive variables related to the use of materials and expectations in relation to learning in the use of AR. Both questionnaires use a 5 or 7-point Likert-type scale [33].

Student motivation is measured in more than 50% of the studies analyzed (*n* = 10). Various authors [6,29,34] used the “Instructional Material Motivational Survey” (IMMS). On the other hand, Nørgård et al. (2019) [35] measured self-efficacy and motivation with a selection of 12 questions through an adapted version of the Printritch instrument (1991) [36]; while Agudelo et al. (2019) used an ad hoc survey [25]. Since AR uses 3D objects, various studies evaluated the spatial visual abilities of the participants through the “Mental Rotation Test” (MRT) [31,34,37].

Other studies used various instruments to measure other aspects, such as the degree of acceptance of AR technology [6] or the cognitive load used in the teaching-learning process [30]. To measure knowledge, questionnaires validated by experts or pre-post ad hoc questionnaires were used [24,27,30,31,34,35,37,38].

#### 3.2.2. Specific Psychopedagogical Outcomes of the Studies

Following the levels described in training programs evaluation, we present the outcome of the reviewed studies regarding the Motivational aspects and satisfaction with the experiences, the learning outcomes and the results of the programs in terms of benefits of AR training in Health Sciences.

##### Influence of AR on Motivational Aspects

Student motivation and involvement towards learning was analyzed in several studies (*n* = 10). In seven of them, students who experimented with AR showed a higher level of motivation compared to those who used other methodologies [6,25,26,27,29,33,39]. In two of them, no significant differences were found in student motivation towards learning [34,35]. According to Vaughn et al. (2016), increasing realism in clinical simulation environments increases motivation [26], thereby improving learning. Barmaki et al. (2019) concluded that their AR system facilitated active learning and improves the involvement of students in the training experience [38].

Only four studies addressed student satisfaction. Specifically, Medicine [29] and Physiotherapy [10] students showed great satisfaction when using an AR-based mobile application to study human anatomy. In the case of Khan et al. (2019) this satisfaction was higher than when the students used the traditional methodology based on notes [29]. This situation also occurred in Nursing students in a laboratory practice that increased simulation realism [11]. Medicine students’ satisfaction also improves by increasing the perception of realism of a clinical simulation through the use of AR, which could enhance understanding of the topic, motivation and student performance [27].

##### Learning Outcomes

(a) Academic performance

Several studies (*n* = 6) measured the academic performance previously and after the AR experience of the students to find out the influence of AR on learning outcomes. In fiveall of them, students that used AR-based methodology obtained significantly higher scores on post-intervention knowledge tests [24,27,30,34,37,38]. Thus, these studies supported that AR experiences enhanced the long-term retention of knowledge about chronic wounds diagnosis [24], and human anatomy [27,31,37], including the musculoskeletal system [38] and neuroanatomy [34].

(b) Acquisition of clinical competencies

AR improved clinical decision-making skills in the treatment of chronic wounds in Nursing students [24]. In the same line, the research by Quqandi et al. (2018) concluded that AR facilitates the learning of basic skills in clinical nursing practices [11], and Rochlen et al. (2017), showed that AR improved technical skills in the placement of a central venous catheter [12].

(c) Acquisition of cognitive skills

Agudelo et al. (2019) underlined the excellent assessment that Phonoaudiology students make on the use of a methodology based on AR, contributing to the construction of learning and collaborative work [25]. These results were in line with those obtained by Cabero et al. (2018) and Kugelmann et al. (2018), in which the students positively valued the use of AR as support for the teaching-learning of human anatomy, providing student-centered learning and facilitating three-dimensional understanding of human anatomy [6,39].

Only two studies measured the cognitive load used by students in the acquisition of knowledge [30,34]. In both, those groups that used AR as a teaching methodology experienced a lower cognitive load.

Regarding spatial visual skills, two articles showed that the stereoscopic effect provided by AR improves spatial understanding. This occurred especially in those students with low visual spatial capacity, making it a useful tool to facilitate the comprehension of anatomical structures [31,37].

##### Benefits of Engaging on AR Experiences

There are no studies that have focused on the benefits of training experiences using AR beyond the training programmes, that is, in the actual development of health sciences professionals. However, some studies allow us to infer the specific benefits of undertaking such experiences by comparing participants included in AR training programmes with control groups, that is, using other learning methodologies. In this sense, the use of AR in the teaching-learning processes has been pointed to provide multiple benefits, such as a higher performance in the diagnosis of chronic wounds [24], a more effective learning and a lower cognitive load in the study of neuroanatomy [30], higher scores in knowledge tests [27,33]. AR increases student engagement [28], self-confidence [35] and facilitates the three-dimensional understanding of the human anatomy [31,34,37,38]. On the contrary, Henssen et al. (2019) and Nørgard et al. (2019) did not find increased academic performance in the AR group respect to the control group [34,35].

#### 3.2.3. Pros and Cons of Using AR in Health Science Education

The authors of the articles selected in this research detailed multiple advantages and disadvantages of AR as a teaching methodology. These aspects could be considered when planning how AR will be used as an element to support the teaching-learning process.

Among the pros can be highlighted that AR enhancement the real world because allows the incorporation of virtual objects into a real environment, which allows to enrich reality, not replace it [24]. Through this combination of real and virtual objects, learning environments can be created that promote student immersion in the teaching content and offer visualizations that would not otherwise be possible. This enables to increase the understanding of such invisible or difficult to visualize objects or phenomena in a real environment [29]. Its cost is low and the access is easy, AR teaching contents can be reproduced through mobile devices, allowing the use of this technology for educational purposes without an added cost, since both tablets and smartphones are electronic devices widely used by the educational community [28,30]. AR stimulates collaborative learning and self-learning [25,27,30,33] and facilitates learning and skills acquisition, regardless of user experience level [12]. AR also allows in-depth study of an anatomical structure by allowing the disassembly of its parts, offering a three-dimensional visualization with which the student can interact, for example, allowing its parts to be disassembled, in a virtual sense, and put back together. [6,28,31,34].

Regarding the cons, the difficulty in using and handling some devices can be underlined. Participants in the experience of Bogomolova et al. (2020), used smart glasses to visualize the AR teaching content. They pointed out difficulties in adjusting the glasses, instability of the projected image and the need to keep the head still, among others [31]. Nørgård et al. (2019) also used smartglasses as a display device activated by gestures that had to be very precise, causing difficulties among the participants [35]. Likewise, Henssen et al. (2019) mentioned that AR did not allow tactile feedback in the study of brain anatomy [34]. In addition, the participants found difficulties in handling the materials necessary to visualize the contents, concluding that these difficulties in interaction negatively affected the learning process.

AR is a new technology little implemented in education and its incorporation as a teaching technology tool is recent, so research on this topic is novel but scarce [6]. In addition, ignorance about its use can cause difficulties in its handling by students [26]. The findings of Moro et al. (2017) showed adverse effects reported by students who used AR in a teaching intervention. The analyzed symptoms were classified into general (discomfort, fatigue, boredom, nausea or headache) and ocular (eye fatigue, blurred vision or double vision) [28].

Sometimes AR has a high cost of implementation. Bogomolova et al. (2020) used the highest level of AR, augmented vision, with smartglasses that provide stereoscopic 3D vision. They identified as a disadvantage the high cost of the development of the experience, which, on the other hand, facilitates the learning of students with low spatial visual skills, an improvement compared to other cheaper AR levels (for example, mobile devices that allow monoscopic vision) [31]. In addition, a lack of content developers was found. One of the main obstacles to the integration of AR technology in classrooms is the development of 3D multimedia content. This technology is still under-utilized because there are not enough experts available to generate AR-based interactive teaching materials [30].

## 4. Discussion

The aims of the study were to identify and summarize the AR interventions used in higher education in health sciences, the impact of AR on educational outcomes and skills, as well as the main advantages, disadvantages, and challenges during the teaching-learning process.

The use of AR in health sciences degrees has increased in the last few years. This has been largely due to the massive use of mobile electronic devices by students, which greatly facilitates the implementation of this educational tool in the classroom [28,30,40]. In 52.6% (*n* = 10) of the articles included in this systematic review, smartphones or tablets were mostly used to interact with AR content. Despite the boom experienced in recent years, the use of AR as an innovative technological educational tool is still quite limited, despite the great advantages that have been found. This is mainly due to the reluctance of teachers, the lack of training and means to generate 3D content. Perhaps this is due to the scarcity of research that demonstrates the efficacy and effectiveness of this technology, since most of the studies analyzed have a small sample size in a single institution, which makes it difficult for the data to be generalizable.

Most of the research included in this review on the usefulness of AR was developed in the field of Anatomy (*n* = 13). Evidence shows that the curricular competences of this course are clearly favored by AR, especially in students with low visual spatial abilities [31,37]. This is so because allows the visualization of different anatomical structures in 3D as well as the same structure from multiple perspectives [6,28,34]. This is in line with research carried out in other fields, such as Fine Arts or Geography, where AR facilitates the understanding of 3D space and improves students’ spatial orientation skills [41]. It can also be extended to Chemistry or Biology, where 3D models are used for learning [42].

In relation to motivational aspects, they have been extensively studied in selected articles. Of the total number of articles that evaluate motivation and involvement (*n* = 10), in most of them (*n* = 8) AR significantly increased both. In very few (*n* = 2), no significant differences were found associated to the use of an AR-based teaching methodology. In the case of Henssen et al. (2019) these findings could be due to the fact that AR is compared with the use of cadaveric material, which is considered the gold standard in the study of anatomy [34]. Therefore, we agree with other authors that motivation plays a determining role in the learning process [43,44]. Likewise, the findings of our review agree with the results of other authors in various fields and educational levels, such as Engineering, Education Sciences or Arts, where AR has been extremely useful to increase the motivation of students towards learning [4,45,46,47,48].

Likewise, from the analysis of 19 studies, various aspects emerged that we synthesize as follows. It allows the development of technical skills and clinical decision-making, regardless of the level of training of individuals. Therefore, AR constitutes a useful tool in the teaching-learning process of individuals with a heterogeneous level of knowledge [11,12,24,40]. It makes simulation environments more realistic, generating new interactive learning spaces that facilitate autonomous and collaborative learning. Thus, this technology promotes more flexible learning, since AR mobile applications allow access to information anywhere and at any time [6,12,25,26,27,33]. It improves spatial understanding, especially in those students with low visual spatial ability [31,37,39]. Also, it improves student motivation and satisfaction, enhancing learning outcomes while reducing cognitive load [6,10,24,25,26,27,29,30,33,34,39].

Due to all the foregoing, we can state that we are at the birth of the educational use of AR. At the same time, its great potential as a teaching instrument stands out, since it offers numerous advantages compared to few limitations in the teaching-learning processes.

### 4.1. Relevance to Clinical and Teaching-Learning Practice

The articles included in this review highlight the usefulness of RA in various teaching areas of clinical practice in Higher Education in Health Sciences. Rochlen et al. (2017) report on the usefulness of this technology to improve the skills of students and professionals in the performance of a medical technique consisting of the canalization of a central venous catheter [12]. It was also useful to improve decision-making in relation to the diagnosis and treatment of chronic wounds [24], to develop skills for home visits [40], to increase the confidence and competence of Nursing students in learning basic clinical skills in the field of clinical training [11], or to increase realism in a clinical simulation environment where students provide nursing care to a manikin [26].

This review provides information on the most used applications for the use of AR, which might help with decision-making and instructional planning in Health Sciences. Even though there are different AR software, some of them are no longer available. This short obsolescence could difficult a long-term use and contribute to teaching overload when having to redesign the didactic materials in a new application.

### 4.2. Strengths and Limitations

The use of AR has increased in the last decade. This systematic review has been done through a multidisciplinary search strategy in three languages, synthesizing and interpreting the findings of the last few years. Thus, it provides information for future research and implementation of educational innovation.

Regarding limitations, the most frequent was the small sample size, which requires caution when interpreting the results and drawing conclusions. In addition, negative results may have been missed due to publication bias. Therefore, more studies are needed to address this issue with more representative and multicenter samples. In this way, it could be studied in greater depth to what extent AR modifies academic performance in different educational contexts at the international level. In the same sense, no studies have tried to contrast the long-term effects in participants’ professional performance or benefits for healthcare units/patients. Due to the nature of this review (pedagogical outcomes) no prior protocol was recorded. More research with standardized instruments and protocols are necessary to perform meta-analysis. Considering the heterogeneity regarding methodologies of the studies, a meta-analysis could not be performed.

## 5. Conclusions

According to this systematic review, AR used as an educational technological tool in university studies in Health Sciences improves the teaching-learning process by influencing it in a multidimensional way.

The use of AR in higher education in the field of Health Sciences reduces the cognitive load and increases the motivation and satisfaction of the students. Its use as a learning support tool also improves spatial understanding and promotes autonomous learning, in line with the guidelines of the European Higher Education Area. Given that AR provides clinical simulation environments with greater realism, we can conclude that the use of this technology in Health Sciences is especially useful in those courses with a large component of 3D vision during the teaching-learning process. However, guidance on how to make effective use of AR and more developers that support the teaching process are needed, together with the promotion of its use through, for example, competitive projects or awareness of higher education institutions towards this technology.

Thanks to the massive use of smartphones among the university community and the free availability of some applications, the teaching use of this technology is being democratized with multiple advantages in the teaching-learning processes.

## Figures and Tables

**Table 1:** Example CINAHL (Ebsco) search.

(MH “Augmented Reality”) AND (MH “Students, Health Occupations+”) OR ((“augmented reality”) AND (“higher education *” OR “university * education *”)) TI OR ((“augmented reality”) AND (“higher education *” OR “university * education *”)) AB

Limited to English, Spanish and Portuguese results. Timespan 2014–2020.

**Table 2:** Data abstraction.

Author(s) & (Year) & Country	Academic Degree & Academic Year & Course & Sample Size	Aims	Measuring Instruments	Results	MMAT Rating
Bogomolova et al. (2020) The Netherlands	Medicine 1st and 2nd year Anatomy *N* = 58 students	To evaluate the effectiveness of stereoscopic visualization of AR and the effect of spatial vision skills on learning.	Mental Rotation Test (MRT), consisting of 30 questions validated by experts to assess knowledge of anatomy. A self-reported questionnaire to evaluate the learning experience (study time, perception of the knowledge acquired, degree of satisfaction with the materials used, etc.)	Significant differences were found in the learning effect between the groups that used 2D teaching material and those that used the 3D stereoscopic vision of AR among the students with low spatial visual abilities measured through the MRT. Group members who viewed the teaching content using AR stereoscopic vision technology enjoyed the session more than those who viewed it in 2D or monoscopic 3D AR.	High
Rochlen et al. (2017) USA	Medicine 3rd and 4th year. Clinical training *N* = 40 students, faculty, and nurse anesthetists.	To evaluate the usefulness of AR in the placement of a central venous catheter	Likert scale online questionnaire. Questions about previous experience in performing the technique, satisfaction with the use of AR and perception about the use of AR in medical procedures.	According to participants, AR technology is realistic, easy to use, fun, and promotes learning. They perceive that AR is useful in improving their skills and that it would be a useful adjunct to medical training.	High
Borges et al. (2016) Brasil	Nursing, Physiotherapy and Medicine Not specified Familiy Health *N* = 135 students	To evaluate the adequacy of teaching materials based on AR games applied to the teaching of home visits	Questionnaire designed *ad hoc*.	AR-based educational games were highly rated by the participants.	Moderate
Henssen et al. (2019) The Netherlands	Medicine 1st year Anatomy *N* = 31 students	To investigate differences in the test score, cognitive load and motivation between the experimental group (AR-based teaching methodology) and the control group (non-AR teaching methodology)	Pre- and post-experience questionnaires with questions on neuroanatomy in control and AR group. MRT. Instructional Materials Motivation Survey (IMMS).	No significant differences in knowledge test scores between both groups were found. There were no significant differences in terms of cognitive load between both groups, although the cognitive load of the group that used AR was lower. There were no significant differences in the different dimensions of the IMMS.	Moderate
Nørgård et al. (2019) Denmark	Medicine Not indicated Anatomy *N* = 110 students	To examine the effectiveness of a teaching methodology with AR on short- and long-term learning compared to a methodology without AR.	Questionnaires before and after the experience with questions about Anatomy of the mediastinum. Adaptation of “Motivated Strategies Learning Questionnaire” (MSLQ).	There were no significant differences between the groups (control and experiemental -AR group) in terms of motivation and test scores. The mean self-efficacy was significantly higher for the group with AR teaching methodology.	High
Khan et al. (2019) South Africa	Medicine Not indicated Anatomy *N* = 78 students	To measure the impact of the AR mobile application on motivation for learning in Health Sciences students.	Modified IMMS. Online open-question survey designed *ad hoc*.	The use of an AR mobile application increased students’ motivation.	High
Bork et al. (2019) Germany	Medicine 1st year Anatomy *N* = 72 students	To quantitatively compare a teaching methodology with AR and a teaching methodology without AR for the learning of Human Anatomy.	Initial knowledge questionnaire. Final knowledge questionnaire with questions other than the initial questionnaire MRT. Survey designed by experts in medical education on perception with the teaching methodology used.	The group that used an AR teaching methodology (experimental group) obtained significantly better results. Results of the MRT were similar in both groups. The AR-based system is considered by students as a valuable addition to the study of Anatomy, increasing spatial understanding.	High
Barmaki et al. (2019) USA	Medicine 2nd year Anatomy *N* = 288 students	To assess the degree of engagement and retention of knowledge of students in a collaborative learning intervention based on AR	Pre- and post- intervention knowledge questionnaire in control and AR group	The interactive AR system significantly improved retention of knowledge and increased the level of engagement	High
Cabero et al. (2018) Spain	Medicine 1st year Anatomy *N* = 50 students	To determine the degree of motivation and acceptance of AR in Medicine students and to evaluate the teaching materials.	IMMS for motivation analysis. The Technology Acceptance Model (TAM). Likert scale questionnaire developed ad hoc to evaluate teaching materials.	The interaction with the AR objects greatly increased motivation of the students. The students showed a high degree of acceptance of this technology. The teaching materials with AR were valued positively.	Moderate
Kugelmann et al. (2018) Germany	Medicine 1st year Anatomy *N* = 880 students	To find out if an educational intervention based on AR can be a valuable addition to the traditional methodology in the teaching-learning of Human Anatomy.	Anonymous and voluntary Likert-type scale questionnaire and open questions.	The educational intervention with AR obtained a positive evaluation of the students, increasing their motivation and considering it beneficial for learning.	High
Quqandi et al. (2018) United Kingdom	Nursing Clinical training Not indicated *N* = 4 students	To assess how mobile AR enhances the self-regulation skills of Nursing students in a clinical training laboratory.	Questionnaire administered before and after the intervention.	The students were satisfied and declared that they enjoyed using mobile AR. Mobile AR increased their confidence and competence in learning basic clinical nursing skills.	Low
Hoang et al. (2017) USA	Physiotherapy Postgraduate Anatomy *N* = 9 students	To know how an immersive technology such as AR can improve the teaching-learning of Physiotherapy.	Questionnaire before and after the intervention. Informal conversations.	AR applied to the teaching-learning of Physiotherapy was valued positively in the analyzed spheres, highlighting satisfaction, fun and understanding of the Anatomy.	Moderate
Moro et al. (2017) Australia	Medicine Not indicated Anatomy *N* = 59 students	To analyze if AR is as effective as the use of tablets in learning the Anatomy	Likert-style questionnaire after the intervention.	There were no significant differences between the two methodologies, but the AR-based methodology provided benefits such as a greater degree of immersion and student participation.	High
Ferrer-Torregrosa et al. (2016) Spain	Medicine, Physiotherapy, Chiropody 1st year Anatomy *N* = 171 students	To assess if a teaching methodology based on AR provides a higher degree of learning than a teaching methodology based on videos and traditional notes when the student uses it through distance learning.	Questionnaire to collect the time dedicated to the experience. Assessment questionnaire on the knowledge acquired through autonomous learning. Closed and validated questionnaire to measure metacognitive variables (attention and motivation, autonomous learning and three-dimensional understanding) in relation to the use of teaching materials and expectations related to learning in the use of AR.	The group with AR teaching methodology devoted more study time to the educational intervention. AR helped students maintain attention and increased student motivation. Regarding the autonomous learning dimension, the evaluations made by the students who used AR were statistically higher than those of the rest of the groups. They considered that AR improved 3D understanding and that it was an ideal complement to the study of Anatomy. Regarding the expectations, a large majority considered that AR-based teaching methodology it is effective to study, that it increases motivation and interest and that their grades would improve if the teachers used it.	Moderate
Vaughn et al. (2016) USA	Nursing Not indicated Clinical training *N* = 12 students	To evaluate if RA increases the students’ perception of realism in a clinical training laboratory.	Online survey based on the “Simulation Design Scale” (SDS) and the “Self-Confidence in Learning Scale” (SCLS).	Most students strongly endorsed that AR improves realism in a clinical simulation, contributes to autonomous problem solving, is motivating, and beneficial for a simulation-based learning environment.	High
Küçüc et al. (2016) Turkey	Medicine 2nd year Anatomy *N* = 70 students	To determine the effect of mobile AR on students’ learning outcomes and cognitive load.	Pre and post academic performance test developed by experts. Cognitive Load Scale.	The experimental research group (Mobile AR) obtained a higher academic performance with a lower cognitive load.	High
Jamali et al. (2015) Australia	Medicine Not indicated Anatomy *N* = 30 students	To describe the process of developing a learning environment based on Mobile AR. To measure the changes in the knowledge, behavior and attitudes of the participants after using the mobile AR-based application.	Knowledge test before and after the intervention in control and experimental research group (AR group). Likert scale questionnaire on the functionality of the mobile application.	Mobile AR increased understanding of content, motivation in the learning process and improved student learning performance.	High
Agudelo et al. (2019) Chile	Speech Therapy Not indicated General Processes of Speech Therapy *N* = 61 students	To describe the perception of students regarding the contribution of AR to the acquisition of skills defined in the curriculum of the course.	Perception survey prepared ad hoc consisting of three dimensions: contributions to training, collaborative learning and motivation with AR.	Good evaluations were obtained in all three dimensions. AR encourages the development of curricular competencies.	High
Jorge et al. (2016) Portugal	Nursing 1st year Chronic wound care *N* = 54 students	To assess if AR improves the development of clinical decision-making skills in relation to the diagnosis and treatment of chronic wounds.	Knowledge tests before and after the intervention consisting of solving practical cases.	AR significantly improved the results of the students in the phase of “diagnosis of chronic wounds”. In the “treatment” phase, no significant differences were found.	High

**Table 3:** Applications, electronic devices and AR levels.

Authors (Year) [Reference]	Application Used to Develop AR Content	AR Display Devices	AR Level ^1^
Jorge et al. (2016)	Autodesk 123D & ViewAR	Mobile devices	2
Jamali et al. (2015)	Unity^®^3D, Vuforia^®^, & Human Anatomy in Mobile Augmented Reality (HuMAR)	Mobile devices	2
Küçüc et al. (2016)	Axiom Neuro 1.0, Neuromatiq 1.0, Anatomy 4D & Aurasma	Mobile devices	2
Vaughn et al. (2016)	Not indicated	Smartglasses	3
Moro et al. (2017)	Unity^®^ & Vuforia^®^	Mobile devices	2
Hoang et al. (2017)	Unity^®^ 3D & Augmented Anatomy	Smartglasses	3
Quqandi et al. (2018)	Unity^®^, Vuforia^®^	Mobile devices	2
Kugelmann et al. (2018)	Not indicated	Screen	2
Cabero et al. (2018)	Layar, Metaio Creator, Metaio SDK, Augment & Aurasma	Mobile devices	2
Barmaki et al. (2019)	CMake, Open Graphics Library & Microsoft Kinect Software Development.	Screen	2
Bork et al. (2019)	Not indicated	Screen	2
Khan et al. (2019)	Anatomy 4D	Mobile devices	2
Nørgård et al. (2019)	Not indicated	Smartglasses	3
Henssen et al. (2019)	Unity^®^ & Grey-Mapp.	Mobile devices	1
Borges et al. (2016)	ARToolKit.	Mobile devices	1
Rochlen et al. (2017)	Unity^®^ Game Engine & Vuforia^®^	Smartglasses	3
Bogomolova et al. (2020)	Dynamic Anatomy	Smartglasses	3
Ferrer Torregrosa et al. (2016)	Not indicated	Mobile devices	2

^1^ AR levels (Reinoso, 2013) [32]: level 0: Hyperlink to the physical world, based on QR codes. level 1: Marker-based AR, the most popular mode of AR. level 2: Markerless AR, based on image recognition or geolocation. level 3: Increased vision. Most advanced level of AR that allows a direct augmented visualization of the environment using smart glasses or contact lenses.

## Data Availability

The data that support the findings of this study are available from the corresponding author upon reasonable request.

## Supplementary Materials

The following are available online at https://www.mdpi.com/article/10.3390/ijerph18084262/s1, Table S1: PRISMA 2009 checklist, Table S2: Quality appraisal of quantitative descriptive studies, Table S3: Quality appraisal of quantitative studies (non-randomized: case-control studies), Table S4: Quality appraisal of quantitative studies (randomized controlled trials), Table S5: Quality appraisal of mixed methods studies.
