# Supplementary material for: A Systematic Review of Augmented Reality in Health Sciences: A Guide to Decision-Making in Higher Education
Source: Int J Environ Res Public Health. 2021 Apr 17;18(8):4262. doi: 10.3390/ijerph18084262 (PMC8073641; doi:10.3390/ijerph18084262)
Supplement: Supplementary file 1 [file ijerph-18-04262-s001.zip › ijerph-1173210-supplementary.pdf]

**Table S1.** PRISMA 2009 Checklist (Moher et al., 2009).

| Section/Topic | # | Checklist Item | Reported on Page, Table or Figure |
|---|---|---|---|
| **TITLE** | | | |
| Title | 1 | Identify the report as a systematic review, meta-analysis, or both. | 1, 3 |
| **ABSTRACT** | | | |
| Structured summary | 2 | Provide a structured summary including, as applicable: background; objectives; data sources; study eligibility criteria, participants, and interventions; study appraisal and synthesis methods; results; limitations; conclusions and implications of key findings; systematic review registration number. | 1 |
| **INTRODUCTION** | | | |
| Rationale | 3 | Describe the rationale for the review in the context of what is already known. | 1, 2 |
| Objectives | 4 | Provide an explicit statement of questions being addressed with reference to participants, interventions, comparisons, outcomes, and study design. | 3 |
| **METHODS** | | | |
| Protocol and registration | 5 | Indicate if a review protocol exists, if and where it can be accessed (e.g., Web address), and, if available, provide registration information including registration number. | 3 |
| Eligibility criteria | 6 | Specify study characteristics and report characteristics used as criteria for eligibility, giving rationale. | 3, 4 |
| Information sources | 7 | Describe all information sources in the search and date last searched. | 3, 4 |
| Search | 8 | Present full electronic search strategy for at least one database, including any limits used, such that it could be repeated. | 3, 4 |
| Study selection | 9 | State the process for selecting studies (screening, eligibility, included in systematic review). | 4, Figure 2 |
| Data collection process | 10 | Describe method of data extraction from reports and any processes for obtaining and confirming data from investigators. | 5 |
| Data items | 11 | List and define all variables for which data were sought and any assumptions and simplifications made. | Table 2 |
| Risk of bias in individual studies | 12 | Describe methods used for assessing risk of bias of individual studies (including specification of whether this was done at the study or outcome level), and how this information is to be used in any data synthesis. | 5 |
| Summary | 13 | State the principal summary measures. | NA |

| | | | |
|---|---|---|---|
| measures | | | |
| Synthesis of results | 14 | Describe the methods of handling data and combining results of studies, if done, including measures of consistency for each meta-analysis. | NA |
| Risk of bias across studies | 15 | Specify any assessment of risk of bias that may affect the cumulative evidence. | 5 |
| Additional analyses | 16 | Describe methods of additional analyses if done, indicating which were pre-specified. | NA |
| **RESULTS** | | | |
| Study selection | 17 | Give numbers of studies screened, assessed for eligibility, and included in the review, with reasons for exclusions at each stage, ideally with a flow diagram. | Figure 2 |
| Study characteristics | 18 | For each study, present characteristics for which data were extracted and provide the citations. | Table 2 |
| Risk of bias within studies | 19 | Present data on risk of bias of each study and, if available, any outcome level assessment (see item 12). | Table 2 |
| Results of individual studies | 20 | For all outcomes considered (benefits or harms), present, for each study: (a) simple summary data for each intervention group (b) effect estimates and confidence intervals, ideally with a forest plot. | NA |
| Synthesis of results | 21 | Present results of each meta-analysis done, including confidence intervals and measures of consistency. | NA |
| Risk of bias across studies | 22 | Present results of any assessment of risk of bias across studies (see Item 15). | 5 |
| Additional analysis | 23 | Give results of additional analyses, if done (e.g., sensitivity or subgroup analyses, meta-regression [see Item 16]). | NA |
| **DISCUSSION** | | | |
| Summary of evidence | 24 | Summarize the main findings including the strength of evidence for each main outcome; consider their relevance to key groups (e.g., healthcare providers, users, and policy makers). | 5-15 |
| Limitations | 25 | Discuss limitations at study and outcome level (e.g., risk of bias), and at review-level (e.g., incomplete retrieval of identified research, reporting bias). | 16, 17 |
| Conclusions | 26 | Provide a general interpretation of the results in the context of other evidence, and implications for future research. | 15-17 |
| **FUNDING** | | | |
| Funding | 27 | Describe sources of funding for the systematic review and other support; role of funders for the systematic review. | 17 |

NA: Not applicable.

**Table S2.** Quality appraisal of quantitative descriptive studies (Hong et al., 2018).

| Mixed Methods Appraisal Tool (MMAT) Criteria | First Author & (Year) | | | | |
|---|---|---|---|---|---|
| | Borges F et al. (2016) [ref?] | Cabero et al. (2018) | Kugelmann et al. (2018) | Hoang et al. (2017) | Agudelo et al. (2019) |
| Clear research question(s) | Yes | Yes | Yes | Yes | Yes |
| Collected data addresses the research questions | Yes | Yes | Yes | Yes | Yes |
| Sampling strategy is relevant to address the research question(s) | Yes | No | Yes | No | Yes |
| Sample is representative of target population | Yes | Yes | Yes | Yes | Yes |
| Measurements are appropriate | No | Yes | Yes | Yes | Yes |
| The risk of nonresponse bias is low | Yes | No | Yes | Yes | Yes |
| The statistical analysis is appropriate to answer the research question | No | Yes | No | No | Yes |

**Table S3.** Quality appraisal of quantitative studies (non-randomized: case-control studies) (Hong et al., 2018).

| Mixed Methods Appraisal Tool (MMAT) Criteria | First Author & Year | | | |
|---|---|---|---|---|
| | Rochlen et al. (2017) | Nørgård et al. 2019. | Jamali et al. 2015. | Jorge el al. 2016 |
| Clear research question(s) | Yes | Yes | Yes | Yes |
| Collected data addresses the research questions | Yes | Yes | Yes | Yes |
| Participants are representative of the target population | Yes | Yes | Yes | Yes |
| Measurements are appropriate regarding both the outcome and intervention (or exposure) | Yes | Yes | Yes | Yes |
| Outcome data are complete | Yes | Yes | Yes | Yes |
| Confounders are accounted for in the design and analysis | No | No | No | No |
| During the study period, the intervention is administered (or exposure occurred) as intended | Yes | Yes | Yes | Yes |

**Table S4.** Quality appraisal of quantitative studies (randomized controlled trials) (Hong et al., 2018).

| Mixed Methods Appraisal Tool (MMAT) Criteria | First Author & (Year) | | | |
|---|---|---|---|---|
| | Bogomolova et al. (2020) | Henssen et al. (2019) | Barmaki et al. (2019) | Ferrer-Torregrosa et al. (2016) |
| Clear research question(s) | Yes | Yes | Yes | Yes |
| Collected data addresses the research questions | Yes | Yes | Yes | Yes |
| Randomization is appropriately performed | Yes | No | Yes | No |
| the groups are comparable at baseline | Yes | Yes | Yes | Yes |
| there are complete outcome data | Yes | Yes | Yes | Yes |
| Outcome assessors are blinded to the intervention provided | No | No | No | No |
| The participants adhere to the assigned intervention | Yes | Yes | Yes | Yes |

**Table S5.** Quality appraisal of mixed methods studies (Hong et al., 2018).

| Mixed Methods Appraisal Tool (MMAT) Criteria | Author(s) & Year | | | | | |
|---|---|---|---|---|---|---|
| | Khan et al. (2019) | Bork et al. (2019) | Quqandi et al. (2018) | Moro et al. (2017) | Vaughn et al. 2016 | Küçüc et al. 2016 |
| Clear research question(s) | Yes | Yes | Yes | Yes | Yes | Yes |
| Collected data addresses the research questions | Yes | Yes | Yes | Yes | Yes | Yes |
| Is there an adequate rationale for using a mixed method design to address the research question? | Yes | Yes | Yes | Yes | Yes | Yes |
| Are the different components of the study effectively integrated to answer the research question? | Yes | Yes | No | Yes | Yes | Yes |
| Are the outputs of the integration of qualitative and quantitative components adequately interpreted? | Yes | Yes | No | No | No | No |
| Are divergences and inconsistencies between quantitative and qualitative results adequately addressed? | Yes | Yes | Yes | Yes | Yes | Yes |
| Do the different components of the study adhere to the quality criteria of each tradition of the methods involved? | Yes | Yes | No | Yes | Yes | Yes |
